# Analysis of the Virus Contamination and Disinfection Effect in Isolation Ward of Patients With COVID-19

**DOI:** 10.3389/fpubh.2020.00486

**Published:** 2020-08-18

**Authors:** Shiyang Zhang, Chuanpeng Wang, Minqiang Lin, Qinsheng Deng, Yuzhen Ye, Zhiyong Li, Lixin Qiu, Zhanxiang Wang

**Affiliations:** The First Affiliated Hospital of Xiamen University, Xiamen, China

**Keywords:** COVID-19, environmental surface, virus contamination, cleaning and disinfection, hand hygiene

## Abstract

The recent outbreak of COVID-19 has infected a large number of patients, increasing the importance of adequate disinfection of the hospital environment. We conducted this study to explore environmental virus contamination and the effect of terminal disinfection in the isolation ward of patients with COVID-19. A swab kit was used to sample various surfaces in the isolation and observation wards using the smear method. The samples were immediately sent to the PCR department of the laboratory for nucleic acid detection of COVID-19. We analyzed 31 high-frequency contact sites in three isolation wards of actively sick patients, of which seven were positive (22.58%, 7/31). Positive sites included the transfer window, bed rail, buffer room door handle, toilet door handle, and toilet faucet. All 55 samples taken from the wards of cured patients and the wards after terminal disinfection were negative. Virus contamination in areas frequently touched by patients in the isolation ward was high, so the awareness of correct disinfection must be increased. Use of 1,000–2,000 mg/L chlorine-containing disinfectant in the isolation ward was effective.

## Introduction

Since the outbreak of COVID-19 in Wuhan, in December 2019 (1–3), more than 86,000 cases have been confirmed in China as of the end of 19 July 2020. The government have taken urgent measures, including declaring it a category B infectious disease and managing it as a category A infectious disease, the most stringent classification of infectious diseases (4). Suspected and confirmed cases of COVID-19 are diagnosed and treated in a specific hospital, which poses a challenge in terms of preventing cross-infection. The environment or objects in hospitals, once contaminated by patients' blood and body fluids (5), can be a new source of infection. If hands and clothing become contaminated, medical staff and other patients could be infected, causing hospital-acquired infections and even an infection outbreak. Therefore, it is important to effectively clean and disinfect environmental surfaces in hospitals (6, 7). To effectively guide disinfection of the hospital isolation ward, staff from the hospital infection management department went to the isolation ward of patients newly-diagnosed with COVID-19. Samples were taken from surfaces to investigate environmental virus contamination and the result of virus inactivation by the current disinfection method.

## Research Design and Methods

### Research Design

We visited the designated COVID-19 treatment hospital in Xiamen, China from 25 February to 27 February 2020 and used the smear method to sample objects in the COVID-19 isolation wards. The sampling time was 2–4 h after disinfection, during which time patients moved about the ward freely. After the actively sick patients were transferred out of the ward, terminal disinfection was performed immediately. Further samples were taken 30 min after terminal disinfection was completed, to investigate whether the current disinfection method can eradicate all viruses on surfaces within the isolation ward of patients with COVID-19. Samples were also taken 2–4 h after disinfection from a ward of patients who had recovered from COVID-19.

### Patient Diagnosis and Release Isolation Standard

According to the requirements of the COVID-19 Diagnosis and Treatment Program (Sixth Edition) (8), the respiratory specimens of patients were all positive for the detection of novel coronavirus nucleic acid by RT-PCR. The criteria for releasing the patients from the isolation are: 1. the patient's temperature is normal for more than 3 days; 2. respiratory symptoms have improved significantly; 3. pulmonary imaging shows significant improvement in exudative lesions in the acute phase; 4. Consecutive negative nucleic acid detection of respiratory specimens (across an interval of at least 24 h). To reduce the false-negative rate of the nucleic acid test, Xiamen hospital tests patients with a negative nucleic acid test three consecutive times across a sampling interval of at least 24 h, and then transfers them to the observation ward in the hospital for 14 days.

### Sampling Method

A virus sampling swab kit was used in the isolation and observation wards to assess viral contamination in the environment. Following correct hand hygiene, we used virus sampling swabs to wipe the surface of places with high-frequency contact in the isolation ward (9, 10). The sampling area was 200–400 cm^2^ on a broad surface, adjusted according to the size of the object surface, and the entire surface was wiped when it was <100 cm^2^. The temperature in the room at the time of sampling was around 20°C. After sampling, the swab was put into sample storage solution and labeled. The specimens were sealed, the external surfaces disinfected and the sample was immediately transferred to the PCR room of the hospital laboratory for virus nucleic acid detection.

### Environmental Cleaning and Disinfection Methods

The daily cleaning and disinfection method in the isolation ward is as follows: a cleaner wearing adequate personal protective equipment enters the isolation ward *via* a dedicated passage for medical staff. They use a 1,000–2,000 mg/L chlorine-containing disinfectant to wipe and disinfect the environmental surfaces at least twice a day. Air disinfection involves a plasma dynamic air disinfection machine, which absorbs and filters the dust in the air and also filters out microorganisms to reduce the risk of aerosol transmission of the coronavirus. Terminal disinfection was performed after patients were transferred out. This method involved an 80–120 mg/L hypochlorous acid spray for air disinfection. A dose of 10–20 ml/m^3^ was atomized *via* a high-speed fan into tiny particles of <20 μm and sprayed evenly in the air to ensure it came into contact with microbial particles. A window was opened for ventilation after 60 min. Surfaces were wiped with a 1,000–2,000 mg/L chlorine-containing disinfectant. If textiles such as clothes or bed sheets needed to be reused, they were sterilized by circulating steam or boiling for 30 min; or soaked in 500 mg/L chlorine-containing disinfectant for 30 min, and then cleaned as usual. High-value textiles were cleaned using ethylene oxide.

### Instruments and Reagents

Viral nucleic acid was detected using the new coronavirus nucleic acid detection kit (Xiamen Anpuli Biological Engineering Co., Ltd) as per the RT-PCR fluorescent probe method (11).

## Results

### Environmental Surface Virus Contamination in the Isolation Ward of Actively Sick Patients With COVID-19

**Figure 1:**
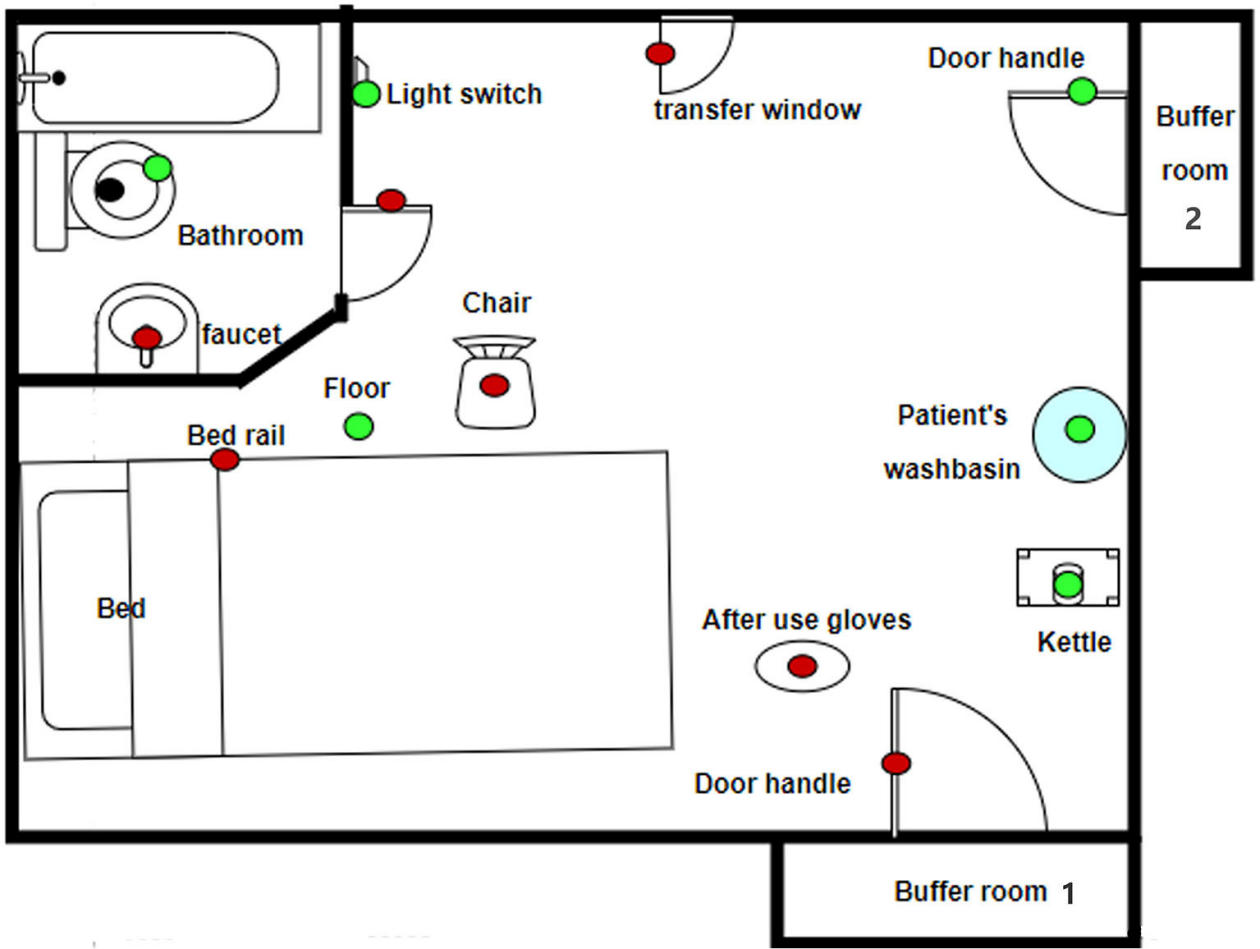
Virus contamination in a typical isolation ward (green is negative, red is positive).

A total of 31 samples were collected from three isolation wards of patients actively sick with COVID-19. Of these samples, seven were positive (22.58%, 7/31). The positive specimens were sampled from the transfer window, bedrail, chair, one buffer room door handle, toilet door handles, toilet faucet and used gloves. The results are shown in Table 1 and Figure 1.

**Table 1:** Sampling results in isolation wards of actively sick patients with COVID-19.

**Sampling site**	**Sample size**	**Positive sample**	**Positive rate**
Transfer window	3	1	33.33%
Bed rail	3	1	33.33%
Bedside table	3	0	0.00%
Light switch	2	0	0.00%
Chair	1	1	100.00%
Floor	3	0	0.00%
Patient's washbasin	1	0	0.00%
Buffer room door handles	3	1	33.33%
Toilet door handles	3	1	33.33%
Toilet faucet	3	1	33.33%
Kettle	2	0	0.00%
Toilet surface	3	0	0.00%
Gloves after use	1	1	100.00%
Total	31	7	22.58%

### Environmental Surface Virus Contamination in the Isolation Ward of Cured Patients

A total of 31 samples were collected from three observation wards, including transfer windows, buffer room door handles, toilet faucets, bed rails, bedside tables, and so on. The samples were all negative for nucleic acid testing (Table 2), which means no virus was found in the ward of the cured patients.

**Table 2:** Sampling results in isolation wards of cured patients with COVID-19 and the isolation wards after terminal disinfection.

**Sampling site**	**Isolation ward of cured patients**			**Wards after terminal disinfection**		
	**Sample size**	**Positive sample**	**Rate**	**Sample size**	**Positive sample**	**Rate**
Transfer window	2	0	0.00%	2	0	0.00%
Bed rail	2	0	0.00%	2	0	0.00%
Bedside table	3	0	0.00%	2	0	0.00%
Light switch	2	0	0.00%	1	0	0.00%
Chair	1	0	0.00%	2	0	0.00%
Floor	3	0	0.00%	2	0	0.00%
Buffer room door handles	3	0	0.00%	2	0	0.00%
Toilet door handles	3	0	0.00%	2	0	0.00%
Toilet faucet	3	0	0.00%	2	0	0.00%
Kettle	2	0	0.00%	1	0	0.00%
Toilet surface	3	0	0.00%	2	0	0.00%
Wall	1	0	0.00%	1	0	0.00%
Mattress	1	0	0.00%	1	0	0.00%
Equipment belt	1	0	0.00%	1	0	0.00%
Gloves After use	1	0	0.00%	1	0	0.00%
Total	31	0	0.00%	24	0	0.00%

### Virus Inactivation After Terminal Disinfection in Isolation Ward of Actively Sick Patients of COVID-19

We sampled environmental surfaces in two isolation wards after terminal disinfection and obtained 24 samples, all of which were negative (Table 2). This means no virus contamination was found on environmental surfaces in the isolation ward after terminal disinfection.

## Discussion

Current research (8) shows that the main source of coronavirus infection is patients, including those who are asymptomatic, *via* droplet transmission and close contact. High concentrations of aerosols in relatively closed environments could lead to aerosol transmission (11, 12). According to research on coronavirus resistance, common disinfectants such as chlorine-containing disinfectants, 75% ethanol, hydrogen peroxide, and hypochlorous acid have good coronavirus inactivation effects (13, 14). Chlorine-containing disinfectants should be chosen if the objects are corrosion- resistant, but 75% ethanol should be chosen first when objects are non-resistant (15).

Patients with COVID-19 can directly contaminate surfaces in the surrounding environment through coughing and can contaminate their clothing or hands through improper cough etiquette. Contaminated clothing or hands can infect environmental surfaces. To guide the thorough infection of the surrounding environment, it was necessary to sample surfaces from an isolation ward of patients with COVID-19. Sampling was also performed to investigate the effect of terminal disinfection on virus inactivation.

A total of 31 samples were collected from the areas frequently contacted by actively sick patients, and the positive rate was 22.58% (7/31). The distribution of positive specimens was mainly on the toilet door handle, buffer room door handle, toilet faucet, and transfer window. Since these are all frequently touched, this indicates that the high-risk areas were those most likely to be contaminated through touching. This finding was supported by the positive sample collected from used gloves, indicating the higher risk of transmission *via* contaminated hands. In addition, the chairs and bed rails may have been contaminated by the patient's clothing or hands.

The door handle of buffer room one was also positive. This was used by medical and cleaning staff to leave the ward and was barely touched by patients, indicating that not all medical staff or cleaners followed correct hand hygiene before leaving the ward. Therefore, we conducted hand hygiene training for all staff. Previous studies found similar results. Ong et al. (16) also demonstrated the survival of coronaviruses on the surfaces of a patient ward toilet and hand basin; Ye et al. (17) demonstrated the survival of coronaviruses on the surfaces of isolation ward door handles and used gloves; Kampf et al. (18) showed that the new coronavirus can survive on inanimate surfaces for a certain period of time (glass or plastic for up to 9 days). These studies show that contaminated objects may become a new source of infection, increasing the risk of cross-infection in the hospital.

Of the 31 specimens collected from three observation wards of recovered patients, no positive samples were found. This indicates that the hospital's protocol is successful. The protocol involves transferring the patient to an observation ward only after three consecutive negative nucleic acid tests performed 24 h apart and using 1,000–2,000 mg/L chlorine-containing disinfectant to wipe and disinfect the environmental surfaces. Similarly, samples were negative from the isolation ward after terminal disinfection, indicating that the current terminal disinfection method meets the requirements for virus inactivation. In order to reduce the risk of cross-infection in the hospital, 1,000–2,000 mg/L chlorine-containing disinfectant can be used for environmental cleaning and disinfection in short-term hospitalization, especially to strengthen the cleaning and disinfection of the high-frequency contact parts of the patients' hands.

There are certain shortcomings in this study, however. For example, as most cases have been cured, the sample size is limited and the contamination from patients with severe COVID-19 has not been investigated. No research has been conducted on other disinfectants and no research has been conducted on the disinfection effect of cloth. In the future, other regions or medical institutions with confirmed patients can explore this area further to provide more theoretical support to guide future disinfection strategies.

In summary, virus contamination in the isolation ward of actively sick patients with COVID-19 is common, especially in areas frequently touched by patients. Such high-frequency contact areas should be disinfected more frequently by enhancing the awareness of disinfection whenever necessary. The current terminal disinfection method of hypochlorous acid in the air combined with chlorine disinfectant on surfaces can meet the requirements of virus inactivation.

## Data Availability Statement

All datasets generated for this study are included in the article/supplementary material.

## Ethics Statement

This study had been reviewed and approved by the Ethics Committee of the First Affiliated Hospital of Xiamen University (Ethics Review Number 2020-003).

## Author Contributions

ZW and SZ conceived and designed the experiments. CW, ML, YY, ZL, and LQ performed the experiments. CW, QD, and SZ analyzed the data and wrote the manuscript. ZW and ML critically revised the manuscript. All authors read and approved the final version of the manuscript.

## Conflict of Interest

The authors declare that the research was conducted in the absence of any commercial or financial relationships that could be construed as a potential conflict of interest.
